# Impurity profiling of paracetamol toxic impurities in pharmaceutical combination with ibuprofen and chlorzoxazone using HPLC and TLC densitometric methods

**DOI:** 10.1186/s13065-025-01466-6

**Published:** 2025-04-24

**Authors:** Israa A. Wahba, Said A. Hassan, Ahmed S. Fayed, Sally S. El-Mosallamy

**Affiliations:** 1 Pharmaceutical Analytical Chemistry Department, College of Pharmaceutical Sciences and Drug Manufacturing, Misr University for Science & Technology, 6th of October City, Giza, Egypt; 2 Pharmaceutical Analytical Chemistry Department, Faculty of Pharmacy, Cairo University, Kasr-El Aini Street, Cairo, 11562 Egypt

**Keywords:** Chlorzoxazone, HPLC, Ibuprofen, Impurity profiling, Paracetamol, TLC densitometry

## Abstract

This work presents two methods for the simultaneous determination of ibuprofen (IBU), paracetamol (PAR), and chlorzoxazone (CHZ) in the presence of three PAR impurities: *p*-aminophenol (PAP), *p*-nitrophenol (PNP), and *p*-chloroacetanilide (PCA). Furthermore, both methods attempt to quantify these hazardous impurities. The first method is a thin layer chromatography densitometric method (TLC), where separation was achieved on silica gel 60 F_254_ plates using a mobile phase consisting of chloroform, toluene, ethanol, and ammonia (7.0: 1.0: 1.6: 0.2, by volume). Densitometric detection was performed at 220.0 nm. The second method is a high-performance liquid chromatographic method (HPLC), in which the analytes were separated on an Xterra C8 column (150 × 4.6 mm, 5 µm) using an isocratic mobile phase of acetonitrile and phosphate buffer (pH 7.5) in a 30:70 (v/v) ratio. The UV detector was set at 220.0 nm, and the flow rate was maintained at 0.7 mL/min. Both methods were validated following ICH guidelines and successfully applied to the determination of IBU, PAR, and CHZ in their commercial tablet formulations. A statistical comparison with a previously reported method confirmed no discernible differences in the results, demonstrating the reliability and accuracy of the proposed techniques.

**Supplementary Information:**

The online version contains supplementary material available at 10.1186/s13065-025-01466-6.

## Introduction

“Impurity profiling” refers to a set of analytical techniques used to detect, characterize, identify, and quantify both organic and inorganic impurities, as well as residual solvents, in pharmaceutical dosage forms and bulk drugs [1]. This process plays a crucial role in synthetic drug research and the gram-scale development of novel compounds for pharmacological evaluation, ultimately facilitating the production of bulk pharmaceuticals. Various analytical techniques, including spectroscopy and electrochemical methods, have been employed for stability testing and impurity profiling of pharmaceuticals [2–8]. However, chromatographic techniques remain the most widely used in this domain due to their versatility and precision [9–12]. The ICH guidelines provide specifications to ensure drug and product purity within the pharmaceutical industry. These guidelines are periodically updated to reflect advancements in relevant fields. The ICH Q1A(R2) guideline focuses on long-term and accelerated stability studies that can be performed on new pharmaceutical substances and products, focusing on factors that may affect safety, efficacy, and quality during storage [13]. Additionally, it highlights the importance of stress testing to assess potential changes under various conditions. The Q1E guideline provides recommendations for utilizing stability data to support the development of new drug substances and products [14]. The Q3A(R2) guideline used to regulate the organic impurities in new pharmaceutical products [15]. Meanwhile, the S2(R1) guideline underscores the need for thorough evaluation of genotoxic impurities, given their potential to cause harm even at extremely low concentrations [16]. Together, these guidelines provide a comprehensive framework to ensure the safety, quality, and stability of pharmaceutical products throughout their lifecycle.

**Fig. 1:**
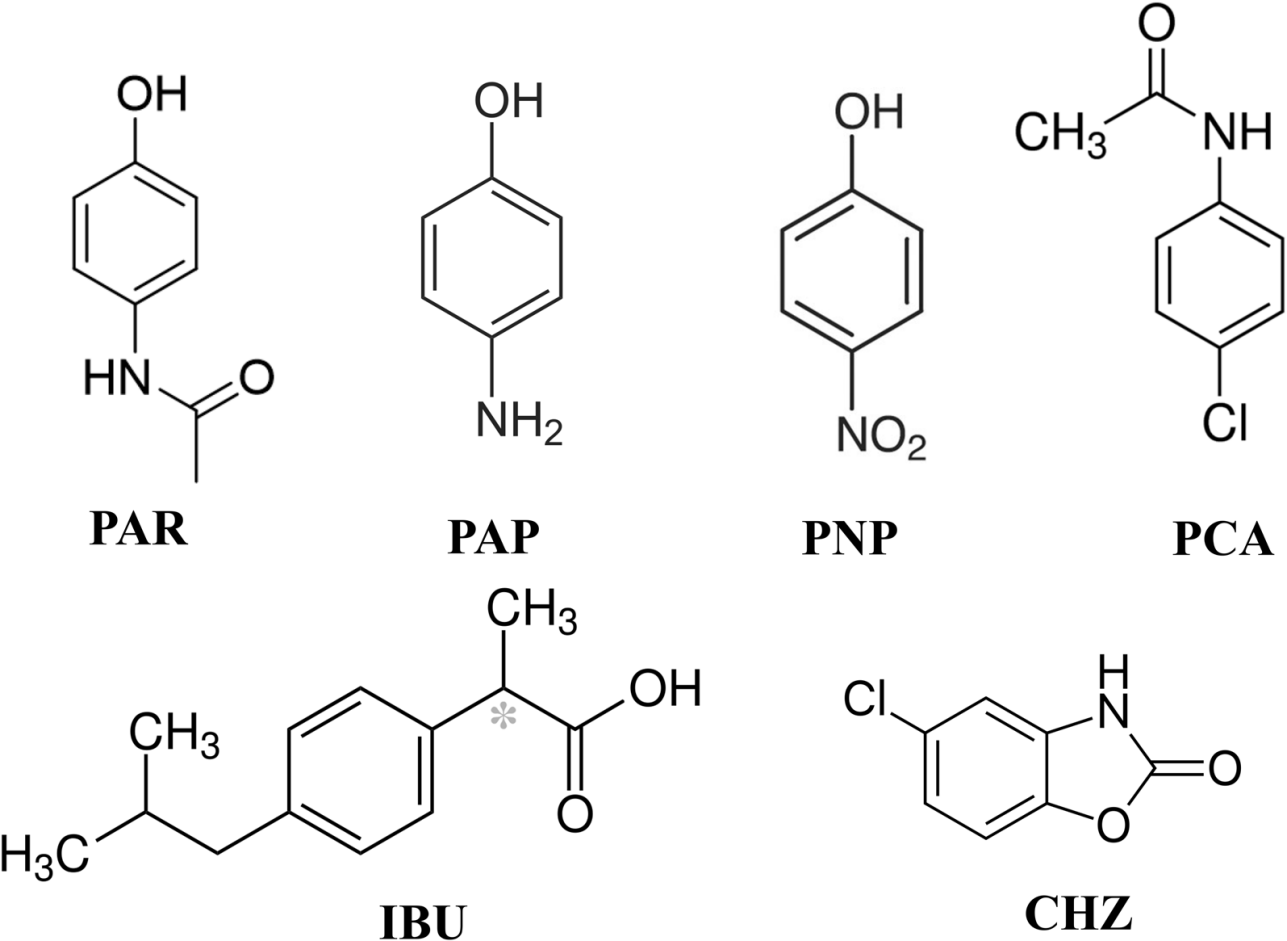
Structural formulae for the compounds under study

Ibuprofen (IBU) (Fig. 1), chemically known as 2-(4-Isobutylphenyl) propanoic acid [17], is an over-the-counter non-narcotic analgesic, anti-inflammatory and antipyretic drug [18]. Paracetamol (PAR) (Fig. 1), chemically known as 4-hydroxyacetanilide [17], is the most common analgesic antipyretic medicament used widely to alleviate several types of pain and to treat fever [19]. Chlorzoxazone (CHZ) (Fig. 1), is chemically named as 5-Chloro-2-benzoxazolone [17]. CHZ is a muscle relaxant drug with mild sedative properties used in a variety of musculoskeletal conditions [20]. It acts by blocking nerve impulses or sensations of pain sent to subcortical areas of the brain [21].

Three main related substances for PAR are included in the present study: 4-aminophenol (PAP), 4-nitrophenol (PNP), 4-chloroacetanilide (PCA) (Fig. 1). PAP, which is defined as impurity K in BP, is the main co-existing impurity in PAR that can arise from either synthesis or degradation processes [22]. It has teratogenic, hepatotoxic, and nephrotoxic effects therefore, it needs to be monitored to ensure that it does not exceed 50 ppm in PAR drug substance and 1000 ppm (0.1%) in pharmaceutical formulations [23]. PNP is categorized in BP as non-specified impurity F, meaning that its concentration cannot be more than 500 ppm in drug substance and 2500 ppm in formulations [22]. PNP concentrations need to be kept under control since it can cause methemoglobinemia [24, 25]. PCA is classified as PAR impurity J in BP and its concentration in drug substances and pharmaceutical formulations must be strictly regulated, not to exceed 10 ppm [22]. It has nephrotoxic and hepatotoxic effects that may cause hemolysis and possess irritating properties that may cause harm to the skin and eyes [26].

Although several analytical methods have been developed for the analysis of these drugs in different formulations [27–31], only a few methods have been reported for their simultaneous determination. These include spectrophotometric [32, 33] and liquid chromatographic techniques [34, 35] ones. To the best of our knowledge, no existing method has been reported for the simultaneous determination of the investigated drugs along with the three PAR impurities (PAP, PNP and PCA).

The aim of this work was directed to establish and validate simple, rapid, and sensitive HPLC and TLC densitometric methods for impurity profiling of PAR along with three of its toxic impurities (PAP, PNP and PCA) in combination with IBU and CHZ.

## Experimental

### Instruments

For TLC, the sample was spotted with a Camag microsyringe (100 μL) at autosampler (CAMAG, Muttenz, Switzerland). TLC Plate was scanned using a Camag TLC scanner 35/N/30319 fitted with winCATS software. A UV lamp (Desaga, Germany) with a short wavelength that emits at 254.0 nm is employed. Aluminum sheets (10 × 20 cm) precoated with silica gel GF_254_ (0.25 mm thickness) for thin-layer chromatography were utilized (Merck, Darmstadt, Germany).

The HPLC system consisted of a Waters Alliance 2695 instrument (Waters, USA) equipped with a quaternary pump and an autosampler injector for chromatographic separation. Separation was carried out using a Waters Xterra C8 column (150 × 4.6 mm, 5 µm), while a Waters 2996 photodiode array detector was employed for compound detection. The pH of the mobile phase was adjusted via an AD1030 pH meter (ADWA, Romania).

### Materials and reagents

#### Pure standard

The three investigated drugs were kindly provided by EVA Pharma-Egypt. Their purity was evaluated by their official methods [22] and was equal to 100.37% ± 1.04, 100.06% ± 0.95 and 99.53% ± 0.89 for IBU, PAR and CHZ, respectively. PAP, PNP, and PCA were provided by Sigma Aldrich (Darmstadt, Germany).

#### Pharmaceutical dosage form

Flexon® MR tablet was manufactured by Aristo Pharmaceuticals Pvt Ltd, India. Each tablet was labeled to contain 400.0 mg ibuprofen, 325.0 mg paracetamol and 250.0 mg chlorzoxazone.

#### Chemicals and reagents

Methanol, ethanol, acetonitrile, and chloroform of HPLC grade were purchased from Merck (Germany). Analytical grade solvents included toluene and ammonia (Adwic, Egypt). Disodium hydrogen phosphate (Oxford, India) orthophosphoric acid, ethylene diamine and double distilled deionized water (Otsuka Cairo, Egypt) were of analytical grade. Phosphate buffer (pH 7.5; 0.03 M) was prepared by dissolving 4.26 g of disodium hydrogen phosphate in double distilled water, and the volume was adjusted to 1000.0 mL. Following that, orthophosphoric acid or ethylene diamine (Biotech, Egypt) were used to adjust the pH [17].

### Solutions

#### Stock standard solutions for TLC and HPLC methods

Stock standard Solutions of 5.0 mg/mL and 1.0 mg/mL for TLC and HPLC methods, respectively, were prepared in methanol for the six compounds.

#### Working standard solutions for HPLC

Working standard solutions of the six cited components (100.0 µg/mL) were prepared in methanol from their respective stock solutions (1.0 mg/mL).

### Chromatographic conditions

For TLC, separation was accomplished on TLC aluminum sheet coated with silica gel 60 F254 plates (10 × 20 cm). The developing system consisted of chloroform: toluene: ethanol: ammonia (7.0: 1.0: 1.6: 0.2, by volume). Samples were spotted as separate compact bands 15.0 mm from the bottom edge of the plates, with 6.0 mm band width. Plates were developed in an ascending manner over a distance of 80.0 mm approximately in a glass chromatographic tank, that was already saturated with the developing system for 60.0 min at room temperature. The developed plates were air dried and then scanned at 220.0 nm with a scanning rate of 20.0 mm/s.

For HPLC, acetonitrile: phosphate buffer (pH = 7.5) (30.0: 70.0, v/v) was used as a mobile phase. Mobile phase was filtered through a 0.45 µm membrane filter and degassed ultrasonically for 30.0 min. Chromatographic separation was accomplished on Xterra C8 column (150 × 4.6 mm, 5 µm) that was conditioned for 30.0 min before sample was injected. Flow rate 0.7 mL/min was kept constant throughout the analysis and UV–vis detector was set at 220.0 nm. The injection volume was 50.0 μL, and all measurements were conducted at room temperature.

### Procedures

#### Construction of the calibration curves

For TLC, aliquots equivalent to 1.0–25.0 mg of IBU, 0.5–20.0 mg of PAP, 1.0–25.0 mg of PAR, 0.5–15.0 mg of PNP, 1.0–20.0 mg of CHZ and 0.5–15.0 mg of PCA were precisely transferred from their stock solutions (5.0 mg/mL) into six different sets of 10-mL volumetric flasks then the volumes were completed to the mark with methanol. Ten microliters from each solution were applied onto the TLC plates using CAMAG Linomat auto-sampler with 100 µL micro-syringe, then analyzed according to the chromatographic conditions previously described. Calibration curves were constructed by relating the integrated peak area to the corresponding concentration of each component and the regression equations were computed.

For HPLC, aliquots equivalent to 10.0–500.0 μg IBU, 2.0–100.0 μg PAP, 10.0–500.0 μg PAR, 2.0–100.0 μg PNP, 10.0–500.0 μg CHZ and 2.0–100.0 μg PCA were accurately transferred from their respective working solutions (100.0 µg/mL) into six series of 10-mL volumetric flasks and the volumes were completed to the mark with the mobile phase. Separation was performed as previously mentioned under chromatographic conditions. Calibration curves representing the relationship between the peak area and the corresponding concentration of each drug were plotted and linear regression equations were computed.

#### Assay of laboratory prepared mixtures.

Different aliquots of the six components were transferred from their stock or working standard solutions into a set of 10-mL volumetric flasks to prepare laboratory prepared mixtures in different ratios. The volume of each solution was adjusted to the mark with methanol or mobile phase for TLC or HPLC, respectively. Concentrations of each component were ascertained using the corresponding regression equation.

#### Application to pharmaceutical dosage form

Ten Flexon® MR tablets were accurately weighed and finely powdered. A portion of the powdered sample, equivalent to 400.0 mg of IBU, 325.0 mg of PAR, and 250.0 mg of CHZ, was transferred to a 100-mL beaker. The sample was sonicated in 30.0 mL methanol for 20.0 min and then filtered into a 100-mL volumetric flask. The residue was washed three times with 10.0 mL of methanol per wash, and the final volume was adjusted to 100 mL using the same solvent. For TLC analysis, appropriate dilutions were prepared in methanol to achieve final concentrations of 800 μg/mL IBU, 650 μg/mL PAR, and 500 μg/mL CHZ. A 10 µL aliquot was spotted onto TLC plates, resulting in the following concentrations: 8 μg/band IBU, 6.5 μg/band PAR, and 5 μg/band CHZ. For HPLC analysis, appropriate dilutions were prepared in the mobile phase to obtain final concentrations of 8 μg/mL IBU, 6.5 μg/mL PAR, and 5 μg/mL CHZ. The analysis of the prepared dosage form solution was conducted using the previously described calibration curve construction method for each technique. The concentrations of IBU, PAR, and CHZ in the dosage form solution were determined using the corresponding regression equations.

## Results and discussion

Pharmaceutical products are susceptible to the presence of impurities or degradates that may be generated during synthesis steps or improper storage conditions where PAP, PNP and PCA are examples of impurities that may be present in PAR [31, 36, 37]. These impurities ought to be quantified along with IBU, PAR and CHZ considering that they are toxic and have a harmful effect on human health. The primary objective of the current work was to develop two chromatographic methods for the simultaneous determination of the target compounds and the quantification of potential impurities in pharmaceutical formulations.

### Method development and optimization

#### TLC method

**Fig. 2:**
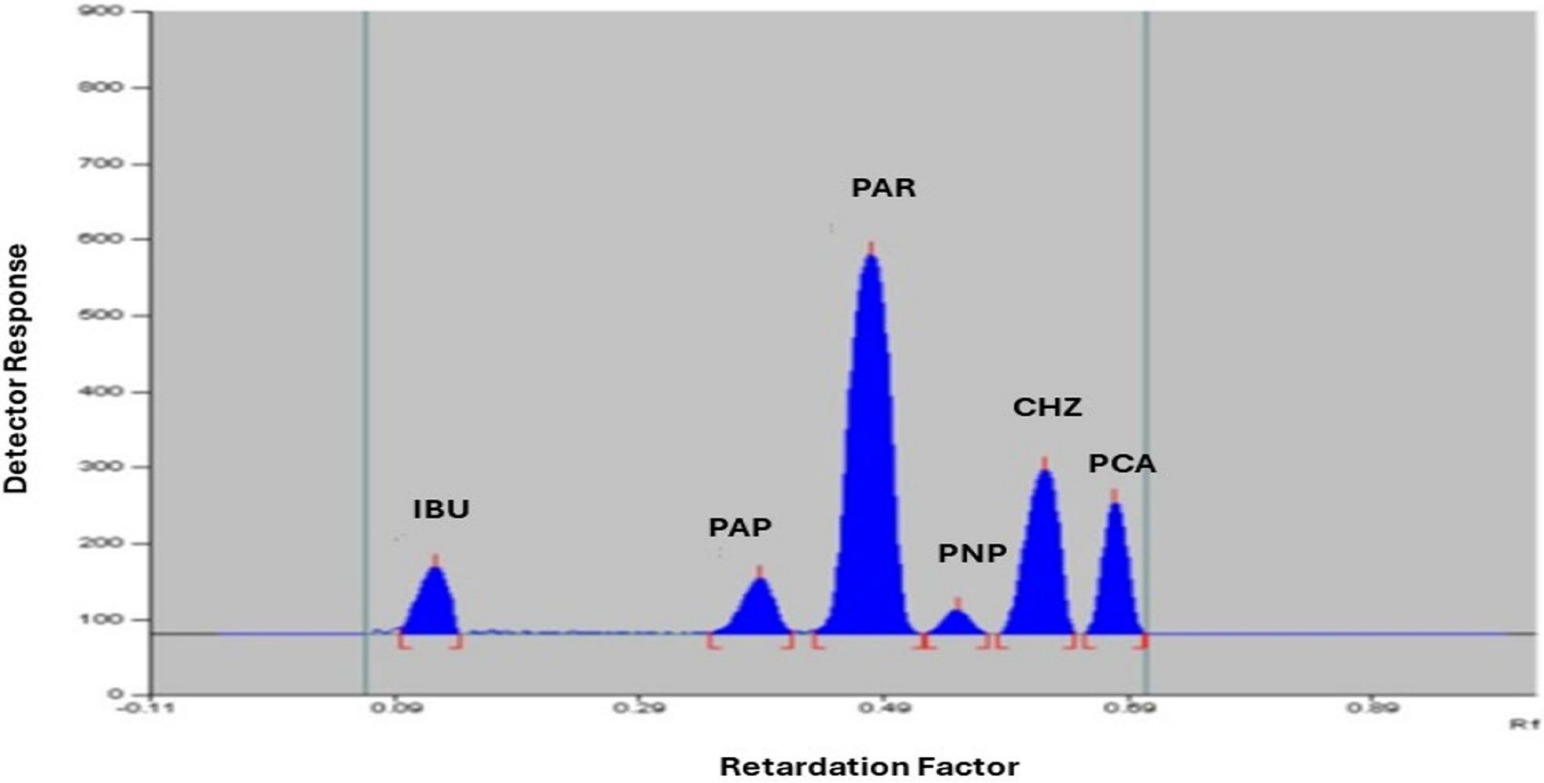
TLC chromatogram of ibuprofen (10.0 μg/band, Rf = 0.12), p-aminophenol (2.0 μg/band, R_f_ = 0.38), paracetamol (10.0 μg/band, R_f_ = 0.48), p-nitrophenol (2.0 μg/band, R_f_ = 0.55), chlorzoxazone (10.0 μg/band, R_f_ = 0.62) and p-chloroacetanilide (2.0 μg/band, R_f_ = 0.68) using a mobile phase of chloroform: toluene: ethanol: ammonia (7.0: 1.0. 1.6: 0.2, by volume) and detection at 220.0 nm

Numerous trials were conducted to establish the optimal chromatographic conditions for achieving adequate separation of the cited components. Several developing systems with different solvent ratios, including ethyl acetate–methanol, toluene–ethyl acetate–acetic acid, and ethanol–chloroform–ammonia, were initially tested, but they failed to provide satisfactory separation. Additional solvent mixtures, such as butanol, ethyl acetate, and formic acid, were also evaluated, but they did not improve the separation. Significant enhancement in separation was achieved by testing different ratios of chloroform, toluene, and ethanol. To further reduce peak tailing and improve peak symmetry, acetic acid, formic acid, and ammonia were incorporated in varying ratios. The best resolution was obtained using a developing system composed of chloroform, toluene, ethanol, and ammonia (7.0:1.0:1.6:0.2, by volume). To optimize sensitivity while minimizing noise, various wavelengths (220.0, 240.0, 254.0, and 260.0 nm) were examined. The best results were obtained at 220.0 nm, where the investigated components exhibited sharp, well-resolved, and symmetrical peaks. As illustrated in Fig. 2, the Rf values in sequence of IBU, PAP, PAR PNP, CHZ, and PCA were 0.12, 0.38, 0.48, 0.55, 0.62 and 0.68, respectively. System Suitability parameters including resolution (Rs), tailing factor (T), capacity factor (k’), selectivity factor (α), column efficiency (N), and height equivalent to theoretical plates (HETP) for the proposed TLC method, were calculated and summarized in Table 1.

**Table 1:** System suitability parameters of TLC-densitometric and HPLC methods

Parameters	TLC -Densitometric method						HPLC						Reference value [39]
	IBU	PAP	PAR	PNP	CHZ	PCA	PAP	PAR	PNP	IBU	CHZ	PCA	
R_f_/t_R_	0.12	0.38	0.48	0.55	0.62	0.68	3.7	5.5	6.9	8.3	9.6	11.0	–
Resolution (Rs)^a^	–	6.56	2.94	2.87	2.05	2.47	–	4.5	4.7	8.2	7.2	5.2	Rs > 2
Selectivity factor (α)^a^	–	4.49	1.50	1.32	1.33	1.30	–	1.14	1.31	1.34	1.19	1.16	α > 1
Tailing factor (T)	0.94	1.06	0.95	1.07	1.06	1.08	0.8	1.0	1.2	1.1	1.0	1.1	T = 0.8–1.2
Capacity factor (K’)	7.33	1.63	1.08	0.82	0.61	0.47	2.1	3.6	4.7	5.9	7.0	8.1	1 < K’ < 10
Column efficiency (N)	–	–	–	–	–	–	2156. 4	3348.9	25,114.2	37,683.8	26,916.5	22,501.5	˃ 2000
Height equivalent to theoretical plate (mm)	–	–	–	–	–	–	0.070	0.045	0.006	0.004	0.006	0.007	The smaller the value, the higher the efficiency

^a^ Chromatographic resolution and selectivity factor are determined between each peak and the one preceding it

#### HPLC method

**Fig. 3:**
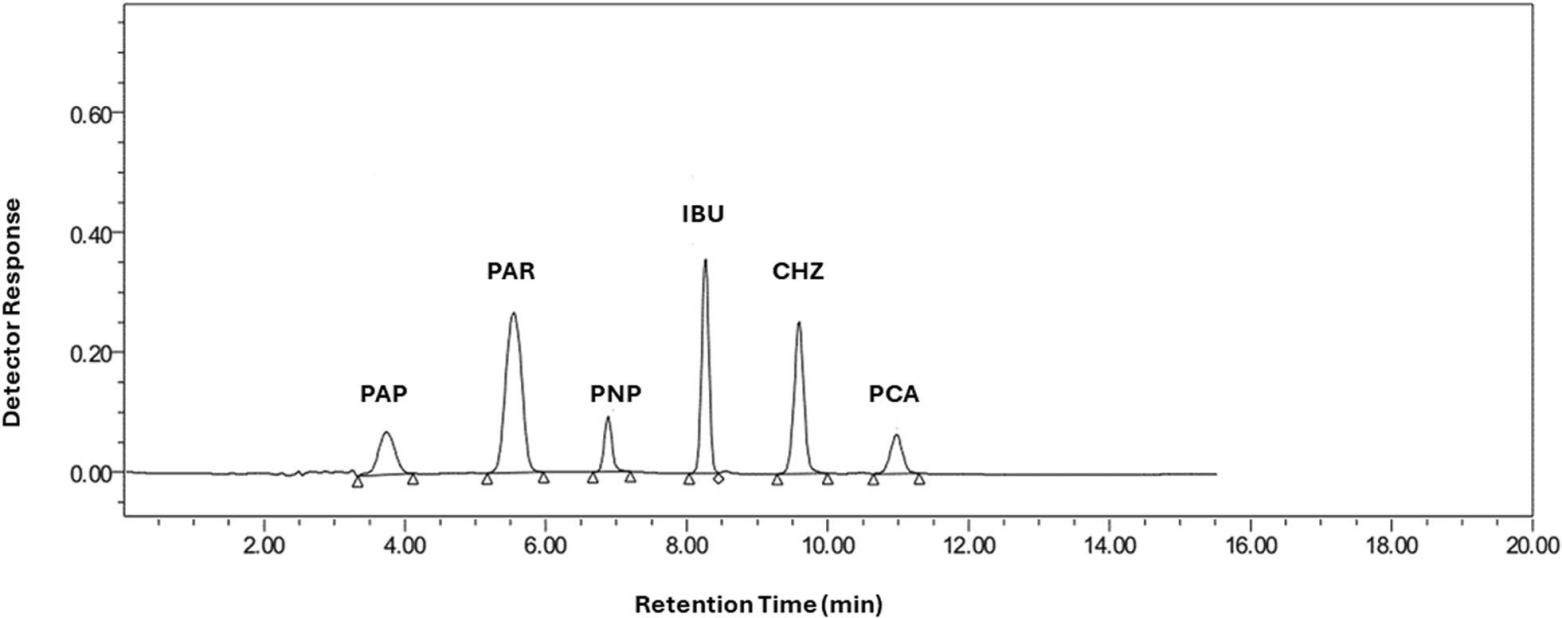
HPLC chromatogram of p-aminophenol (2.0 μg/mL, t_R_ = 3.7), paracetamol (20.0 μg/mL, t_R_ = 5.5), p-nitrophenol (2.0 μg/mL, t_R_ = 6.9), ibuprofen (20.0 μg/mL, t_R_ = 8.3), chlorzoxazone (20.0 μg/mL, t_R_ = 9.6), and p-chloroacetanilide (2.0 μg/mL, t_R_ = 11.0) using an Xterra C8 column (150 × 4.6 mm, 5 µm), mobile phase composed of acetonitrile and buffer (pH = 7.5) at a ratio of 30.0: 70.0 (v/v) with a flow rate of 0.7 mL/min and detection at 220.0 nm

In order to achieve the most optimal separation between the cited drugs and paracetamol impurities, all experimental conditions and variables that could affect the chromatographic separation were investigated. The column, mobile phase composition, pH, flow rate, and wavelength were all adjusted. Three different columns were tried namely, BDS C18, Kromasil Phenyl and Xterra C8. BDS C18 and Kromasil Phenyl columns were unsuitable for the separation owing to delayed peaks. On the other hand, Xterra C8 was the chosen column due to its short run time and good system suitability parameters. Different organic modifiers (methanol and acetonitrile) were tried to give fast separation and enhance the chromatographic separation. Acetonitrile produced sharp peaks with shorter run time, while methanol produced tailed peaks as well as longer run time. Hence, the best organic modifier for achieving optimal chromatographic results was acetonitrile. Similarly, the aqueous phase was optimized, where water and different buffers were tested, and phosphate buffer showed the best chromatographic separation. The ratio of acetonitrile mixture with phosphate buffer was adjusted, where increasing acetonitrile resulted in overlapped peaks, while increasing buffer resulted in longer run time. This can be attributed to the lipophilicity of the compounds under investigation. The pH of the buffer was tested in the range of 3.0 to 9.0 to determine how it affects the peak resolution and retention times of the investigated compounds. It was found that the studied compounds show an overlapped peaks at acidic pH values, whereas basic pH values exhibit undesirable long retention time. Phosphate buffer pH 7.5 was the ideal one for producing well-resolved sharp peaks in a suitable run time. Therefore, isocratic elution was employed using acetonitrile: phosphate buffer (pH = 7.5) at a ratio of 30.0: 70.0 (v/v) to achieve the highest resolution of the adopted components with sharp symmetric peaks. Different flow rates ranging from 0.5 to 1.2 mL/min were tried to study the effect of flow rate on the retention times of the cited compounds. The most suitable flow rate to achieve good separation in an appropriate run time was 0.7 mL/min. Different wavelengths (210.0, 220.0, 230.0, and 240.0 nm) were tried, and it was observed that 220.0 nm showed the best sensitivity for investigation and quantification of the adopted compounds. Optimum separation under the aforementioned chromatographic conditions of the six studied compounds with retention times (t_R_) for PAP, PAR, PNP, IBU, CHZ and PCA were found to be 3.7, 5.5, 6.9, 8.3, 9.6 and 11.0 min, respectively as shown in Fig. 3. To guarantee the performance of the operating system for the adopted chromatographic method, an overall system suitability test was carried out and the results are shown in Table 1.

### Method validation

The two investigated methods were validated in accordance to the ICH guidelines [38].

#### Linearity and range

For TLC method, polynomial correlation was constructed between the integrated peak area and the corresponding concentrations of the six cited components in the ranges of 1.0–25.0 μg/band for IBU and PAR, 1.0–20.0 μg/band for CHZ, 0.5–20.0 μg/band for PAP and 0.5–15.0 μg/band for PNP and PCA. A summary of the regression parameters and their corresponding standard errors are presented in Table 2.

**Table 2:** Validation parameters for determination of IBU, PAP, PAR, PNP, CHZ, and PCA by the proposed methods

Parameter	TLC -Densitometric method						HPLC					
	IBU	PAP	PAR	PNP	CHZ	PCA	PAP	PAR	PNP	IBU	CHZ	PCA
Range	1–25 μg/band	0.5–20 μg/band	1–25 μg/band	0.5–15 μg/band	1–20 μg/band	0.5–15 μg/band	0.2–10 μg/mL	1–50 μg/mL	0.2–10 μg/ mL	1–50 μg/ mL	1–50 μg/ mL	0.2–10 μg/ mL
Slope 1	− 0.4305	− 0.5606	− 0.8544	− 1.8584	− 1.0469	− 1.7476	288,236	216,766	181,532	117,101	124,914	211,516
Slope 2	23.067	23.027	41.22	48.851	42.19	43.655	–	–	–	–	–	–
Intercept	117.65	62.202	93.693	45.994	64.925	58.266	48,994	29,467	− 7227.6	36,568	2978.7	− 17,423
Standard error of slope 1	0.0171	0.439	0.014	0.659	0.040	0.552	1699.832	1311.307	213.283	525.792	183.375	872.961
Standard error of slope 2	0.462	0.021	0.372	0.040	0.845	0.033	–	–	–	–	–	–
Standard error of intercept	2.237	1.061	2.112	2.102	3.732	1.848	5240.654	39,705.414	1182.038	11,000.36	5091.757	3140.686
Correlation coefficient (r)	0.9997	0.9997	0.9999	0.9998	0.9997	0.9997	0.9999	0.9999	1.0000	0.9999	1.0000	0.9999
LOD^a^	0.32 μg/band	0.15 μg/band	0.17 μg/band	0.14 μg/band	0.29 μg/band	0.14 μg/band	0.06 μg/mL	0.30 μg/mL	0.02 μg/mL	0.31 μg/mL	0.13 μg/mL	0.045 μg/mL
LOQ^a^	0.97 μg/band	0.46 μg/band	0.51 μg/band	0.43 μg/band	0.88 μg/band	0.42 μg/band	0.18 μg/mL	0.89 μg/mL	0.07 μg/mL	0.93 μg/mL	0.41 μg/mL	0.15 μg/mL
Accuracy (R% ± SD)^b^	99.47 ± 1.07	98.54 ± 1.19	98.62 ± 0.96	98.94 ± 1.12	99.42 ± 0.98	100.04 ± 1.22	99.74 ± 1.04	99.07 ± 1.33	99.33 ± 0.96	99.74 ± 1.12	100.16 ± 0.83	98.02 ± 0.94
Repeatability (RSD %)^c^	1.41	1.51	1.55	1.36	1.65	1.59	1.39	1.44	1.55	0.97	1.33	1.00
Intermediate precision (RSD %)^d^	1.77	1.76	1.76	1.81	1.90	1.81	1.53	1.77	1.77	1.38	1.57	1.38

^a^ LOD and LOQ were calculated using the following equations: $LOD=3.3\upsigma /\text{S}$ and $LOQ=10\upsigma /\text{S}$ (σ is the residual standard deviation, *S* is the slope)

^b^ Mean (*n* = 3) of three concentrations levels covering the specified range

^c^ Intraday (*n* = 3), three concentrations levels for each compound repeated three times within the same day

^d^ Interday (n = 3), three concentrations levels for each compound repeated three times in three different days

On the other hand, liner relationship was obtained for the HPLC method by plotting peak area versus corresponding concentrations of 1.0–50.0 μg/mL for IBU, PAR, and CHZ and 0.2–10.0 μg/mL for PAP, PNP, and PCA. The regression parameters, with their relative standard errors, are summarized in Table 2.

#### Accuracy

Accuracy of TLC method was validated by analysis of three concentrations levels (5.0, 9.0, 13.0 μg/band for IBU, PAR and CHZ and 5.0, 9.0, 11.0 μg/band for PAP, PNP and PCA) in triplicate. On the other hand, accuracy of HPLC method was ascertained in triplicate at three concentration levels (5.0, 10.0, 20.0 μg/mL for IBU, PAR and CHZ, and 2.0, 4.0, 8.0 μg/mL for PAP, PNP and PCA). All six studied components had satisfactory means of recovery for the investigated methods, which have been assembled into Table 2.

#### Precision

For investigating precision, three chosen concentrations were analyzed in triplicates for assessment of TLC and HPLC methods. Repeatability for both methods were evaluated by analyzing the three concentration levels of the cited components within the same day to investigate intra-day variation. Intermediate precision was performed by analyzing the same concentrations on three consecutive days to assess the inter-day variation. Satisfactory values of relative standard deviation (RSD%) for TLC and HPLC methods were obtained, revealing low deviations and good precision (Table 2).

#### Specificity

The specificity of the proposed methods was evaluated by analyzing several laboratory-prepared mixtures containing varying ratios of the six studied components. The results demonstrated high specificity, as the developed chromatographic methods successfully determined IBU, PAR, and CHZ in the presence of different concentrations of PAR impurities (Table 3). Additionally, satisfactory resolution values exceeding 2 were achieved, confirming the effective separation of the investigated compounds using both the TLC and HPLC methods (Table 1).

**Table 3:** Determination of CHZ, IBU, PAR, PAP, PNP, and PCA in laboratory-prepared mixtures by the proposed methods

TLC -Densitometric method												HPLC method											
Conc (μg/band)						Recovery %^a^						Conc (μg/mL)						Recovery %^a^					
**IBU**	**PAP**	PAR	PNP	CHZ	PCA	IBU	PAP	PAR	PNP	CHZ	PCA	PAP	PAR	PNP	IBU	CHZ	PCA	PAP	PAR	PNP	IBU	CHZ	PCA
9.4	1	8	1	9	1	100.81	101.65	99.67	101.37	101.93	104.18	1	8	1	9.4	9	1	101.63	101.12	101.40	100.88	99.36	100.94
8	2	7.2	2	5	2	101.05	102.55	101.43	102.04	98.27	102.26	2	7.2	2	8	5	2	100.44	99.80	99.23	102.80	102.03	98.50
7	4	6.5	4	9	4	101.54	98.29	98.80	99.18	101.40	102.10	4	6.5	4	7	9	4	99.58	101.89	100.32	100.22	99.92	98.02
6.4	6	6	6	6	6	99.57	101.83	98.23	102.10	101.41	100.82	6	6	6	6.4	6	6	101.93	99.80	101.91	102.00	99.98	99.00
20	8	15	8	18	8	101.74	101.41	98.49	101.91	99.81	100.51	8	15	8	20	18	8	101.03	99.00	100.04	98.46	101.69	100.24
Mean R% ± SD						100.94 ± 0.85	101.15 ± 1.65	99.32 ± 1.30	101.32 ± 1.23	100.56 ± 1.51	101.97 ± 1.45	Mean R% ± RSD						100.92 ± 0.94	100.32 ± 1.16	100.58 ± 1.08	100.87 ± 1.68	100.60 ± 1.19	99.34 ± 1.22

^a^ Average of three determinations

#### Robustness

To assess robustness, several experimental factors were deliberately altered whereas the other parameters were maintained at their optimal values. For both TLC and HPLC, retardation factor/retention time, capacity factor and tailing factor were recorded after each change in the factors. Small changes were allowed in the ratio of ammonia used in mobile phase by a value of 0.2 ± 0.05, time required for saturation with the mobile phase by 60.0 ± 5.0 min and detection wavelength (220.0 ± 5.0 nm). On the other hand, the acetonitrile percentage (30.0 ± 2%), phosphate buffer pH (7.5 ± 0.5) and flow rate (0.7 ± 0.2 mL/min) were intentionally modified for HPLC. The examined parameters for the two adopted methods showed no considerable difference as summarized in (Table 4).

**Table 4:** Robustness assessment of the investigated methods for determination of IBU, PAP, PAR, PNP, CHZ and PCA

	TLC-Densitometric method							HPLC method					
	IBU	PAP	PAR	PNP	CHZ	PCA		PAP	PAR	PNP	IBU	CHZ	PCA
	RSD%^a^							RSD%^a^					
Saturation time 60 ± 5 min							Acetonitrile (%) 30 ± 2%						
Rs^b^	–	1.6	1.4	1.1	1.5	1.4	Rs^b^	–	1.5	1.5	1.0	1.2	0.9
R_F_	1.6	1.5	1.2	1.8	1.6	1.5	tR	1.6	1.2	1.6	1.9	1.2	1.4
K’	1.2	1.9	1.9	1.8	1.4	1.6	K’	1.4	1.6	1.2	1.7	1.4	1.9
T	1.2	0.9	1.2	0.9	1.4	1.1	T	1.9	1.6	1.4	1.6	1.5	1.5
R%	1.9	1.7	1.8	1.5	1.8	1.7	R%	1.2	1.3	1.2	1.5	1.2	1.5
Ammonia content 0.2 ± 0.05 mL							Phosphate buffer pH 7.5 ± 0.5						
Rs^b^	–	1.7	1.6	1.3	1.5	1.8	Rs^b^	–	1.8	1.0	1.2	1.2	0.9
R_F_	1.7	1.5	1.2	1.8	0.9	1.7	tR	1.6	1.5	1.7	1.2	1.7	1.5
K’	1.6	1.6	1.9	1.7	1.5	1.9	K’	1.5	1.6	1.2	1.7	1.7	1.9
T	1.2	1.4	1.0	1.1	1.1	0.9	T	1.7	1.5	1.9	1.8	1.5	1.9
R%	1.6	1.6	1.4	1.9	1.5	1.7	R%	1.5	1.8	1.6	1.4	1.5	1.4
Detector wavelength 220.0 ± 5.0 nm							Flow rate 0.7 ± 0.2 mL/min						
Rs^b^	–	1.8	1.8	1.7	1.8	1.7	Rs^b^	–	1.7	1.4	1.4	1.2	1.3
R_F_	1.7	1.5	1.2	1.9	1.6	1.5	tR	1.3	1.5	1.7	1.3	1.3	1.5
K’	1.4	1.4	1.4	1.7	1.7	1.5	K’	1.2	1.6	1.2	1.0	1.4	1.2
T	1.1	1.4	1.2	1.5	1.1	0.9	T	1.9	1.6	1.4	1.6	1.5	1.7
R%	1.6	1.7	1.6	1.6	1.5	1.8	R%	1.4	1.4	1.8	1.7	1.3	1.8

^a^ RSD% for each system suitability parameter at the three specified conditions

^b^ Resolution is determined between each peak and the one preceding it

### Analysis of dosage form and comparison with reported methods

The proposed chromatographic methods were employed to selectively determine IBU, PAR and CHZ in their pharmaceutical dose form. The results showed that both methods were capable of accurately estimating PAR, IBU, and CHZ without any influence from excipients (Table 5).

**Table 5:** Statistical comparison for the results obtained by the proposed methods and the reported HPLC method [35] for the analysis of IBU, PAR and CHZ in their pharmaceutical preparation

Drug	Parameters						
	Method	Mean	SD	n	Variance	F-test (6.388)^a^	t-test (2.306)^a^
CHZ	TLC	100.45	1.123	5	1.262	1.318	1.799
	HPLC	100.48	1.351	5	1.826	1.097	1.693
	Reported method^b^	99.07	1.290	5	1.664	–	–
IBU	TLC	101.41	1.191	5	1.418	1.846	1.111
	HPLC	100.07	1.321	5	1.744	1.501	0.356
	Reported method^b^	100.41	1.618	5	2.618	–	–
PAR	TLC	100.30	1.421	5	2.018	1.203	1.631
	HPLC	100.15	1.081	5	1.168	2.080	2.00
	Reported method^b^	101.85	1.558	5	2.428	–	–

^a^ Values between parenthesis are corresponding to the theoretical values of t and F (P = 0.05)

^b^ Reported HPLC method using C8 column, acetonitrile: 0.2% triethylamine (50.0: 50.0, v/v), pH adjusted to 3.2 with orthophosphoric acid at flow rate of 1.5 mL/min, detection at 215.0 nm

The student's t-test and the F-test were used to statistically compare the results obtained by the proposed methods for determination of IBU, PAR and CHZ to those obtained by the reported one [35]. There was no discernible difference between the adopted methods and the reported one regarding accuracy and precision (Table 5).

In terms of validation parameters, the proposed HPLC and TLC methods were compared with a previously reported spectrophotometric approach [33]. As shown in Table S1 (Supplementary Materials), the proposed methods demonstrated results comparable to those of the reported method.

## Conclusion

In the present work, two precise, accurate, and selective chromatographic methods were developed for the determination of IBU, PAR, and CHZ in pharmaceutical formulations, as well as for the impurity profiling of PAR. The proposed methods surpass previously published approaches by not only identifying but also quantifying the toxic PAR impurities (PAP, PNP, and PCA). Consequently, these techniques effectively enable both the determination of the target drugs and the assessment of PAR impurity levels. Furthermore, the proposed methods are well-suited for routine analysis of the investigated drugs in bulk powder and pharmaceutical dosage forms, offering a more practical and less complex alternative to existing techniques. Both methods were validated in accordance with ICH guidelines, with the TLC method providing a cost-effective option and the HPLC method offering a more time-efficient approach.

## Supplementary Information

Supplementary Material 1.

## Data Availability

The authors declare that the data supporting the findings of this study are available within the paper and and data sets generated during the current study are available from the corresponding author on reasonable request.

## Abbreviations

BP: British pharmacopoeia
CHZ: Chlorzoxazone
HPLC: High-performance liquid chromatography
IBU: Ibuprofen
ICH: International council for harmonisation
PAR: Paracetamol
PAP: *p*-Aminophenol
PCA: *p*-Chloroacetanilide
PNP: *p*-Nitrophenol
TLC: Thin layer chromatography
