# Supplementary material for: Impurity profiling of paracetamol toxic impurities in pharmaceutical combination with ibuprofen and chlorzoxazone using HPLC and TLC densitometric methods
Source: BMC Chem. 2025 Apr 24;19(1):110. doi: 10.1186/s13065-025-01466-6 (PMC12020172; doi:10.1186/s13065-025-01466-6)
Supplement: Supplementary file 1 — Supplementary Material 1. [file 13065_2025_1466_MOESM1_ESM.pdf]

Supplementary Materials

For

Impurity profiling of paracetamol toxic impurities in pharmaceutical combination with ibuprofen and chlorzoxazone using HPLC and TLC densitometric methods

Israa A. Wahba [a*], Said A. Hassan [b], Ahmed S. Fayed [b], Sally S. El-Mosallamy [b*]

[a] Pharmaceutical Analytical Chemistry Department, College of Pharmaceutical Sciences and Drug Manufacturing, Misr University for Science & Technology, 6th of October City, Giza, Egypt.

[b] Pharmaceutical Analytical Chemistry Department, Faculty of Pharmacy, Cairo University, Kasr-El Aini Street, Cairo 11562, Egypt.

Table S1. Comparison of the validation parameters results obtained by the proposed methods and the reported one (1).

Drug	Validation Parameters					
	Method	Linearity range	Repeatability (RSD %)	Intermediate precision (RSD %)	LOD	LOQ
CHZ	TLC	1-20 µg/band	1.65	1.90	0.29 µg/band	0.88 µg/band
	HPLC	1-50 µg/ mL	1.33	1.57	0.13 µg/ mL	0.41 µg/ mL
	Reported method [a]	0-40 µg/ mL	0.35	0.20	0.07 µg/ml	0.59 µg/ml
IBU	TLC	1-25 µg/band	1.41	1.77	0.32 µg/band	0.97 µg/band
	HPLC	1-50 µg/ mL	0.97	1.38	0.31 µg/ mL	0.93 µg/ mL
	Reported method [a]	0-25 µg/ mL	0.19	0.37	0.12 µg/ml	0.45 µg/ml
PAR	TLC	1-25 µg/band	1.55	1.76	0.17 µg/band	0.51 µg/band
	HPLC	1-50 µg/ mL	1.44	1.77	0.30 µg/ mL	0.89 µg/ mL
	Reported method [a]	0-40 µg/ mL	0.45	0.98	0.42 µg/ml	1.35 µg/ml

[a] Spectrophotometric methods: Q-ratio method and a method based on multicomponent mode of the spectrophotometer.

1. Joshi R, Pawar N, Sawant R, Gaikwad P. Simultaneous estimation of paracetamol, chlorzoxazone and ibuprofen by validated spectrophotometric methods. Analytical Chemistry Letters. 2012;2(2):118–24.
